# Adverse childhood experiences, sarcopenia, and social participation in older adults: a cohort study

**DOI:** 10.1186/s12889-024-18138-0

**Published:** 2024-03-05

**Authors:** Runnian Huang, Yi Li, Chunhua Ma, Rui Ren, Xiaoyue Yuan, Yang Peng, Difei Wang

**Affiliations:** 1 Department of Gerontology and Geriatrics, Shengjing Hospital of China Medical University, 110004 Shenyang, Liaoning China; 2 Department of Health Statistics, School of Public Health, China Medical University, 110122 Shenyang, Liaoning China

**Keywords:** Sarcopenia, Adverse childhood experiences, Social participation, Threat-related adverse childhood experiences, Deprivation-related adverse childhood experiences

## Abstract

**Objectives:**

To examine the relationships between adverse childhood experiences (ACEs) and developing sarcopenia in older adults and the modifying effects of active social participation.

**Methods:**

This prospective cohort study used survey data from the China Health and Retirement Longitudinal Study, including baseline surveys from 2011, follow-up data from 2013, follow-up data from 2015, and information on ACEs from the 2014 Life History Survey. Information concerning 10 ACEs, including five threat-related ACEs and five deprivation-related ACEs before 17 years of age was obtained by questionnaires through face-to-face interviews. Sarcopenia status was assessed according to the Asian Working Group for Sarcopenia 2019 algorithm, consisted of low muscle mass, and low muscle strength, or poor physical performance. The relationship between ACEs, social participation, and sarcopenia was evaluated using Cox proportional hazard regression models.

**Results:**

The study population comprised 6859 older adults in main analyses. Having experienced ≥ 3 ACEs led to an increased 31% risk of developing sarcopenia (hazard ratio [HR]:1.31, 95% confidence interval [CI]:1.10–1.56). Participants having experienced ≥ 2 threat-related ACEs (HR:1.22, 95%CI:1.04–1.43) or deprivation-related ACEs (HR:1.22, 95%CI:1.02–1.46) had a 22% higher risk of developing sarcopenia. Active social participation significantly modified the association between ACEs (p < 0.05), especially threat-related ACEs (p < 0.05), and sarcopenia.

**Conclusions:**

ACEs were associated with the development of sarcopenia; however, social participation had a modifying effect. These findings provide insights for early identification of vulnerable groups, advance intervention timing, and highlight the benefits of promoting active social participation among individuals with sarcopenia who have experienced ACEs.

**Supplementary Information:**

The online version contains supplementary material available at 10.1186/s12889-024-18138-0.

## Introduction

Sarcopenia has been defined as a progressive and generalised skeletal muscle disorder that involves accelerated loss of muscle mass and function and is associated with increased adverse outcomes, including falls, functional decline, frailty, and mortality [1, 2]. Sarcopenia is estimated to affect 10–16% of older adults worldwide, and its prevalence is higher among patients than in the general population [2]. However, sarcopenia also occurs in middle-aged individuals [1, 2], and a previous study has showed that the prevalence of sarcopenia in the age group 50–64 years was 12.7% (95%CI: 10.3–15.7%) [3]. With the increase in life expectancy, sarcopenia results in a more serious socioeconomic burden.

Currently, there is limited research on the risk factors related to sarcopenia. Previous studies have mostly focused on lifestyle and disease factors [2], with few focussing on the association between early life events and sarcopenia in older adults. More than half the global population has experienced adverse childhood experiences (ACEs), and numerous studies have reported that ACEs are associated with lifelong health and healthy behaviours [4, 5]. ACEs are defined as exposure to a series of traumatic events or potentially stressful events during childhood, typically including direct or indirect emotional and physical neglect, abuse, family challenges, and other stress events [4]. A recent cross-sectional study [6] indicates that ACEs are associated with low grip strength and muscle strength in older adults. However, the relationship between ACEs and sarcopenia has not yet been elucidated.

Many researchers have been working on identifying potential effect-modifying factors [7–11] that may alter the risk of adverse health events caused by exposure to ACEs. Current evidence suggests that positive support from the community/society can alleviate the negative effects of ACEs on mental health, physical health, and problem behaviours [8–11]. Contrastingly, living alone and social isolation (limited social participation) are associated with a risk of sarcopenia [12]. However, little is known regarding the modifying role of social participation in the relationship between ACEs and sarcopenia in older adults.

This cohort study aimed to explore the relationship between ACEs and the development of sarcopenia in older adults and the modifying effect of active social participation, using survey data from the China Health and Retirement Longitudinal Study (CHARLS) from 2011 to 2015.

## Methods

### Study design

This cohort study, using survey data from the CHARLS, an ongoing national representative survey targeting individuals over 45 years of age, aimed at providing comprehensive information on older adults for research and policy formulation related to ageing. The detailed research designs and sampling methods have been previously reported [13]. The CHARLS participants were randomly selected using a multistage probability sampling strategy. The baseline survey comprised 17,708 participants from 450 villages and residential communities in 28 provinces across China. Respondents were followed up every two years, and a small share of new participants was recruited in every survey. To date, three follow-up surveys have been conducted in 2013, 2015, and 2018. Information regarding childhood experiences was collected from the 2014 Life History Survey.

Owing to the lack of published physical examination data in 2018, the current study used baseline surveys from 2011, follow-up data from 2013 to 2015, and information on ACEs from the 2014 Life History Survey. Data analysis was performed from 1 July to 30 September 2023. The CHARLS was approved by the Institutional Review Board of Peking University [13]. Written informed consent was obtained from all the participants. This study followed the Strengthening the Reporting of Observational Studies in Epidemiology (STROBE) reporting guidelines. Based on the inclusion and exclusion criteria, the final 6859 participants met the criteria for the main analysis (age range, 45–90 years old). The detailed process for selecting the study participants is listed in the supplementary files (eMethods and eFig. 1).

### Sarcopenia status

Sarcopenia status was assessed according to the Asian Working Group for Sarcopenia 2019 algorithm, which consists of three components: muscle strength, appendicular skeletal muscle mass (ASM), and physical performance [14].

Sarcopenia was diagnosed when low muscle mass, and low muscle strength or poor physical performance were detected. Similar to other sarcopenia-related studies using the CHARLS [15, 16], ASM was estimated in Chinese residents using a validated anthropometric Eqs. [17, 18]. Using dual-energy X-ray absorptiometry (DEXA) as the gold standard, the adjusted R^2^ of the equation model was 0.90 (17, 18). The cutoff points for defining low muscle mass were based on the sex-specific lowest 20% of the height-adjusted muscle mass (ASM/Height^2^) among the study population [15, 17, 18]. This study measured body weight and height using a stadiometer and digital floor scale to the nearest 0.1 cm and 0.1 kg, respectively. Detailed equations and cutoff points can be found in the supplementary files (eMethods).

Handgrip strength (kg) was measured in the dominant and nondominant hands with the participant squeezing a Yuejian TM WL-1000 dynamometer (Nantong Yuejian Physical Measurement Instrument Co., Ltd., Nantong, China) as hard as possible [13]. The cutoff points for low grip strength in men and women were *<* 28 and *<* 18 kg, respectively [14].

Low physical performance was defined in terms of gait speed (< 1 m/s) and the chair stand test (≥ 12s). Further details regarding the definitions of sarcopenia components in the CHARLS have been previously described [19].

### Definition of ACEs

In the 2014 Life History Survey, face-to-face interviews were conducted to collect information on participants’ ACEs before the age of 17. Based on previous studies [7, 11, 20], we extracted ten ACE items and divided them into two dimensions: five threat-related adversities (i.e. physical abuse, household substance abuse, domestic violence, unsafe neighbourhood, and bullying) and five deprivation-related adversities (i.e. emotional neglect, household mental illness, incarcerated household members, parental separation or divorce, and parental death). A detailed definition of ACE indicators can be found in the supplementary files (eMethods and eTable 1), each of which is divided into two categories (0 for absent or 1 for present). We generated a cumulative ACE score by summing all ACE indicators without distinguishing dimensions and divided participants into four categories based on the number of ACEs (0, 1, 2, and ≥ 3). In addition, we divided participation into three categories based on the cumulative scores of the two dimensions of ACEs: threat-related ACEs and deprivation-related ACEs (0, 1, and ≥ 2).

### Definition of social participation

The present study extracted seven types of social participation according to previous literature [21]: (1) interaction with friends, (2) playing Mahjong or other board games, (3) going to sports or social clubs, (4) joining community-related organisations, (5) undertaking voluntary activities, (6) providing help to relatives or others without compensation, and (7) Internet use. Each participant was classified into three categories based on the total number of social participation sessions (none, one, and two or more): 0 for no participation or 1 for participation [21].

### Covariates

Considering that ACEs occur in the early stages of life, and given the previously reported relationship between ACEs and lifestyle behaviours such as smoking and alcohol consumption, as well as various chronic diseases in older adults [7, 22, 23], we considered these factors as mediators rather than confounders. Therefore, to obtain clear effect of ACEs on sarcopenia, we did not include these factors. Instead, based on previous studies, certain possible confounders of the association between ACEs and sarcopenia were selected. These included demographic characteristics (age, sex, and ethnicity) and childhood socioeconomic status (childhood residence and parental education level). Ethnicity was categorised as Han or minority. Childhood residences were divided into cities, towns, and villages. The education level of the parents was defined as the highest educational attainment of either parent for each participant and was further categorised into three groups (illiterate, primary school, middle school and above).

### Statistics

Continuous data were presented as medians with interquartile ranges. Categorical variables are presented as absolute numbers and percentages. Kruskal–Wallis H, Chi-square, or Fisher’s exact tests were used to compare group differences. In the baseline analyses, the associations between ACEs and sarcopenia were estimated using odds ratios (OR) and 95% confidence intervals (CI) with logistic regression models. Based on the year of detecting sarcopenia, 2013 (2 years) or 2015 (4 years), and both death and loss from follow-up considered as censored data, Cox proportional hazard regression models were used to calculate the hazard ratios (HRs) and 95% CIs for the associations between ACEs and the risk of developing sarcopenia during follow-up. We stratified the analyses according to social participation and drew forest and spline plots to determine whether the associations differed according to social participation. The Wald test was used to assess whether the observed relationships were linear or non-linear.

All analyses were conducted on four aspects, namely, all ACEs without distinguishing dimensions (0, 1, 2, and ≥ 3), the number of ACEs as a continuous indicator, threat-related ACEs (0, 1, and ≥ 2) and deprivation-related ACEs (0, 1, and ≥ 2). In the crude models, a single factor was included, and the adjusted models were further controlled for age, sex, ethnicity, childhood residence, and parental education level. To assess the independent associations of threat- and deprivation-related ACEs with the development of sarcopenia, the two dimensions were mutually adjusted in the models. In addition, we included an interaction term of ACEs × social participation in another fully adjusted model to test whether social participation had a modifying effect on the relationship between ACEs and the development of sarcopenia. Furthermore, the causal-steps approach in mediation analysis [24] was used to test the potential mediating effect of social participation on the relationship between ACEs and sarcopenia. Generally, the method requires separate significance testing of the strength of the overall relationship between X and Y, the strength of the relationship between X and the mediator, and the strength of the relationship between mediator and Y adjusted for X. All analyses were performed using R software (version 4.3.1). A 2-tailed p < 0.05 was considered statistically significant.

## Results

### Baseline information

There were 8679 participants (median age, 57; women, 52.3%; men, 47.7%). The prevalence of sarcopenia was 14.6% and, compared to participants without sarcopenia, those with sarcopenia were more likely to be older, of a minority ethnicity, living in the city/town during childhood, with a lower education level of parents, with lower social participation, and with more ACEs (Table 1).

**Table 1:** Characteristics of participants grouped by sarcopenia at baseline

	No	Yes	p
	*N* = 7411	*N* = 1268	
Age	56.0 [49.0;62.0]	66.0 [61.0;72.0]	< 0.001
**Sex**			0.805
Male	3538 (47.7%)	600 (47.3%)	
Female	3873 (52.3%)	668 (52.7%)	
**Ethnicity**			0.001
Han	6908 (93.2%)	1148 (90.5%)	
Minority	503 (6.8%)	120 (9.5%)	
**Childhood residence**			< 0.001
City/town	6759 (91.2%)	1223 (96.5%)	
Village	652 (8.8%)	45 (3.6%)	
**Parental education level**			< 0.001
Illiterate	4151 (56.0%)	863 (68.1%)	
Primary school	2637 (35.6%)	358 (28.2%)	
Middle school and above	623 (8.4%)	47 (3.7%)	
**Social participation**			< 0.001
None	3582 (48.3%)	747 (58.9%)	
One	2476 (33.4%)	401 (31.6%)	
Two and more	1353 (18.3%)	120 (9.5%)	
**ACE group**			< 0.001
0	2195 (29.6%)	312 (24.6%)	
1	2528 (34.1%)	419 (33.0%)	
2	1592 (21.5%)	293 (23.1%)	
≥ 3	1096 (14.8%)	244 (19.2%)	

*Note* The percentages of polytomous variables may not sum up to 100% because of rounding

*Abbreviation* ACE, adverse childhood experience

Of the participants, 28.9%, 34.0%, 21.7%, and 15.4% had experienced 0, 1, 2, and 3 ACEs, respectively. A total of 1422 (16.4%) and 984 (11.3%) participants had experienced two threat- and deprivation-related ACEs, respectively. The prevalence of sarcopenia grouped by ACEs and stratified by social participation is shown in the supplementary files (eFig. 2). The characteristics of the participants grouped by ACEs are shown in the supplementary files (eTables 2–4).

As presented in the supplementary files (eTable 5), in our cross-sectional study, the associations between all ACEs, number of ACEs, and deprivation-related ACEs with the risk of sarcopenia were observed. However, we did not find a relationship between threat-related ACEs and sarcopenia in the cross-sectional analyses. Moreover, increased social participation was associated with a lower risk of sarcopenia (supplementary file, eTable 6).

### Relationship between ACEs, social participation, and developing Sarcopenia

Of the 6859 participants in the longitudinal analyses, 1179 (17.2%) developed sarcopenia within four years of follow-up. The characteristics of participants grouped according to the occurrence of sarcopenia within four years of follow-up are shown in eTable 7. The incidence of sarcopenia grouped by ACEs and stratified by social participation is shown in the supplementary files (eFig. 3). In the Cox proportional hazards regression models, we found that ACEs increased the risk of sarcopenia (Table 2). In all ACEs without distinguishing dimensions, compared to the participants without any ACEs, having experienced ≥ 3 ACEs led to an increased 31% risk of developing sarcopenia (HR:1.31, 95%CI: 1.10–1.56). With more ACEs, the risk of developing sarcopenia increased by 8% (HR: 1.08, 95%CI: 1.03–1.13). Participants having experienced ≥ 2 threat-related ACEs (HR: 1.22, 95%CI: 1.04–1.43) or deprivation-related ACEs (HR: 1.22, 95%CI: 1.02–1.46) had a 22% higher risk of developing sarcopenia.

**Table 2:** Hazard ratios (HRs) of ACEs on developing sarcopenia within four years of follow-up

ACEs	HR (95%CI)	
	Crude model	Adjusted model
**ACE group**		
0	ref	ref
1	1.06 (0.91,1.23)	1.02 (0.88,1.18)
2	1.12 (0.95,1.32)	1.06 (0.90,1.25)
≥ 3	1.35 (1.14,1.61)	1.31 (1.10,1.56)
Number of ACEs	1.08 (1.04,1.14)	1.08 (1.03,1.13)
**Threat-related ACEs**		
0	ref	ref
1	0.97 (0.85,1.11)	0.99 (0.87,1.13)
≥ 2	1.08 (0.93,1.27)	1.22 (1.04,1.43)
**Deprivation-related ACEs**		
0	ref	ref
1	1.15 (1.02,1.30)	1.03 (0.91,1.16)
≥ 2	1.55 (1.30,1.84)	1.22 (1.02,1.46)

*Note* Models were adjusted for age, sex, ethnicity, childhood residence, and educational level of parents, and when distinguishing dimensions, additionally adjusted for the two dimensions

*Abbreviation* ACE, adverse childhood experience; CI, confidence interval

Within four years of follow-up, participants who had engaged in one (HR: 0.85, 95%CI: 0.75–0.97) or two or more (HR: 0.68, 95%CI: 0.57–0.82) types of social participation at baseline had a 15% or 32% lower risk of developing sarcopenia compared to those without any social participation, respectively (supplementary files, eTable 7). With more social participation, the risk of developing sarcopenia decreased by 16% (HR: 0.84, 95%CI: 0.78–0.91).

### Modifying effects of social participation on the relationship between ACEs and developing Sarcopenia

**Fig. 1:**
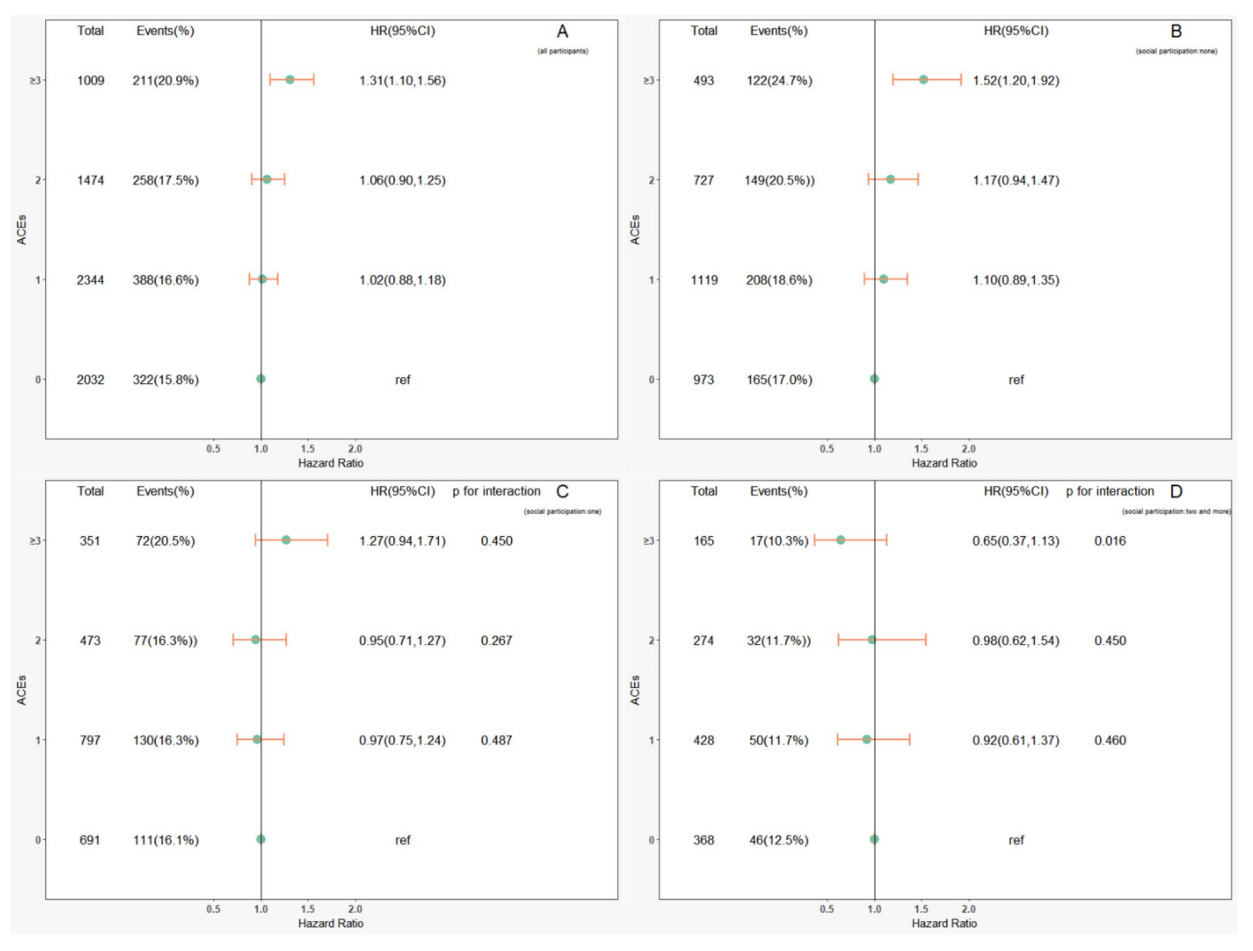
Relationship between adverse childhood experiences (ACEs) and developing sarcopenia by subgroup analysis in terms of social participation within four years of follow-up. (A: all participants; B: social participation = none; C: social participation = one; D: social participation = two and more)

**Fig. 2:**
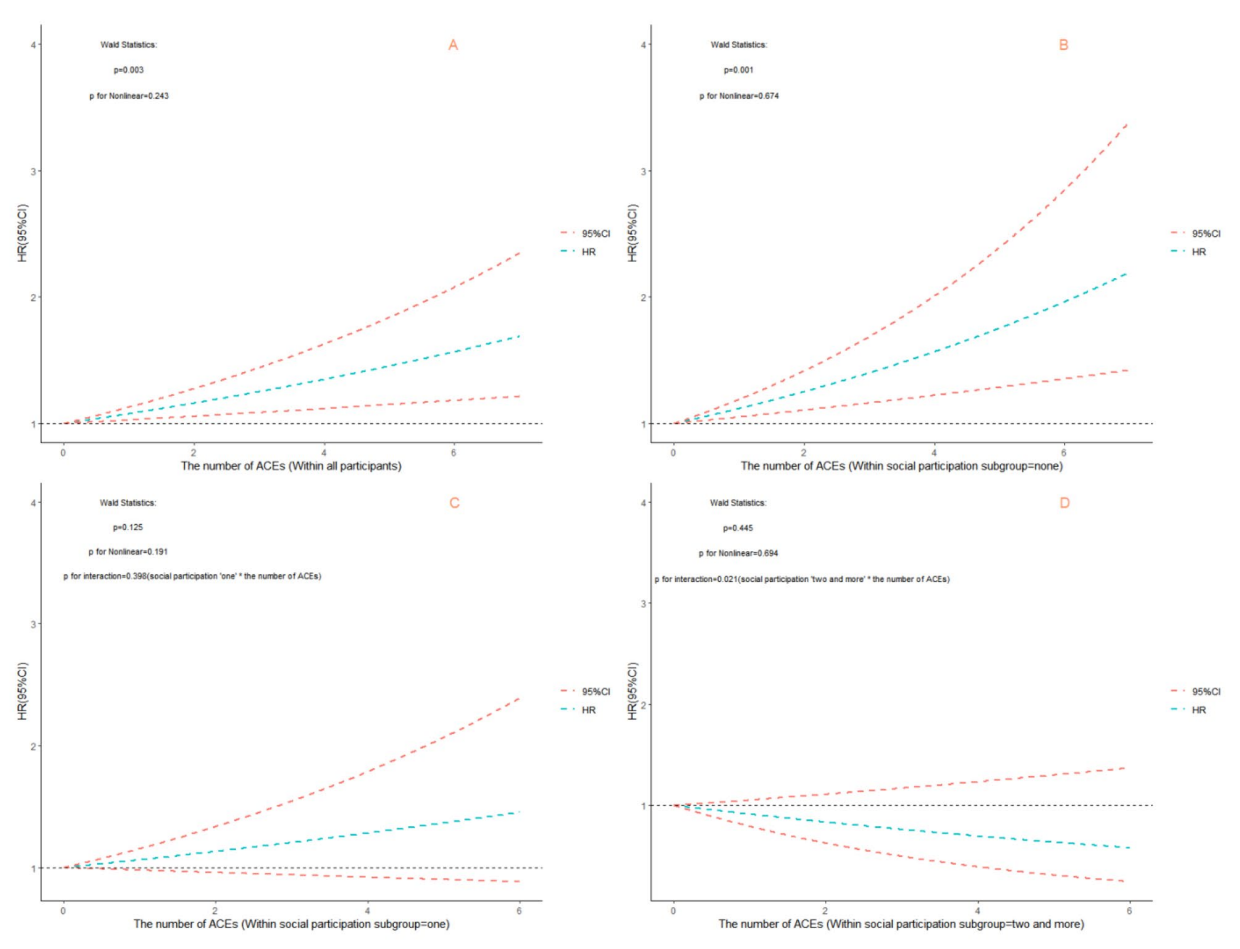
Relationship between the numbers of those with adverse childhood experiences (ACEs) and developing sarcopenia by subgroup in terms of social participation within four years of follow-up. (A: all participants; B: social participation = none; C: social participation = one; D: social participation = two and more)

**Fig. 3:**
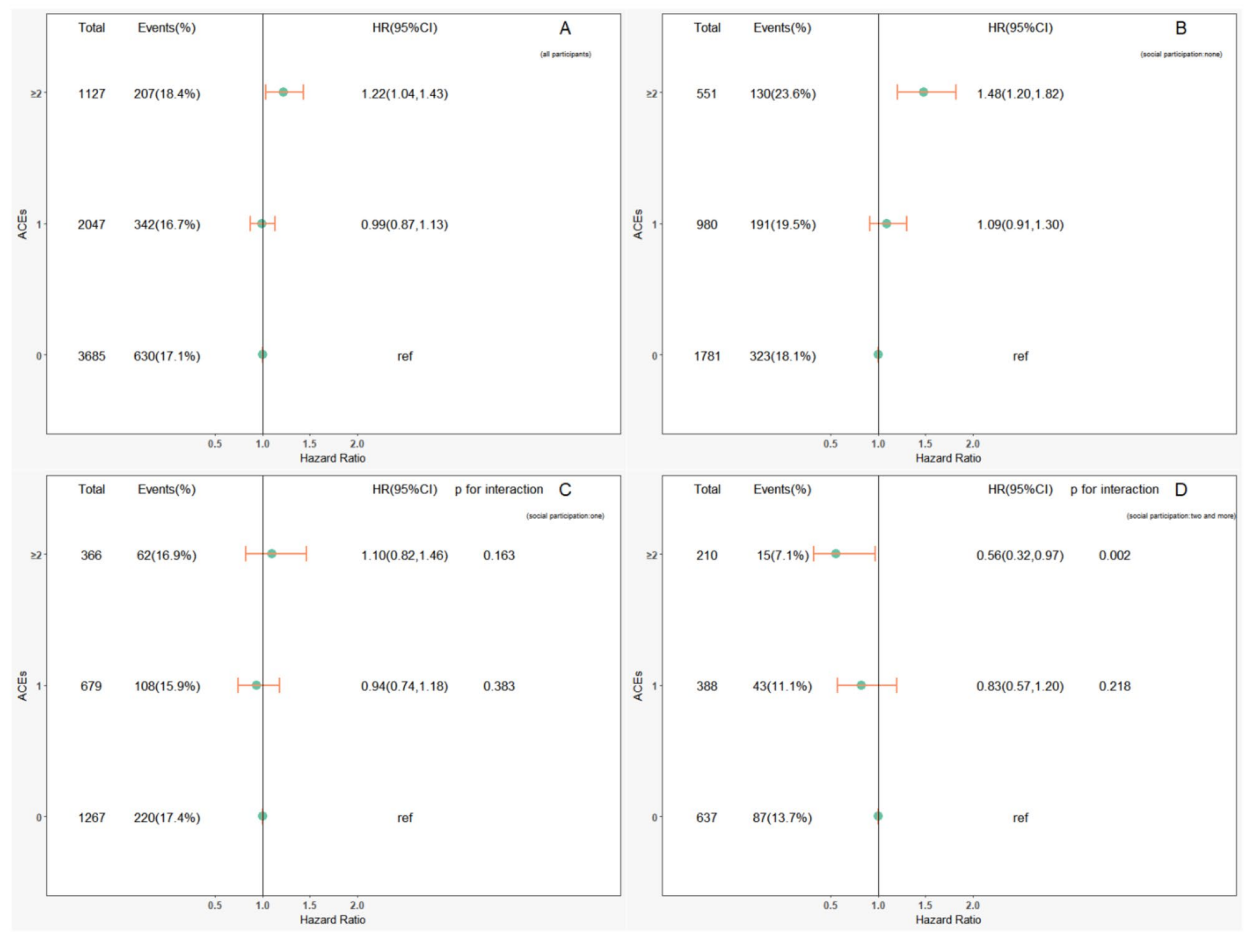
Relationship between threat-related adverse childhood events (ACEs) and developing sarcopenia by subgroup analysis in terms of social participation within four years of follow-up. (A: all participants; B: social participation = none; C: social participation = one; D: social participation = two and more)

Subsequently, through interaction and subgroup analyses, we found that social participation had a modifying effect on the relationship between ACEs and the development of sarcopenia. In all ACEs without distinguishing dimensions, for participants who had engaged in two or more types of social participation at baseline, the effect of having experienced ≥ 3 ACEs on developing sarcopenia decreased (Fig. 1, p for interaction: 0.016). In addition, when treating ACEs as continuous variables, participation in two or more social activities also weakened the negative effects of ACEs on the development of sarcopenia (Fig. 2, p for interaction: 0.021). This modifying effect was the same for threat-related ACEs. Participants who participated in two or more social activities were less affected by the harmful effects of threat-related ACEs on sarcopenia development (Fig. 3, p for interaction: 0.002). However, we did not find any modifying effects of social participation on the relationship between deprivation-related ACEs and the development of sarcopenia (eFig. 4; all p values for interaction: >0.05).

Furthermore, using the causal-steps approach, we did not find a relationship of X (ACEs) and the mediator (social participation), indicating that social participation was not a mediator in the association between ACEs and sarcopenia (supplementary files, eTable 9).

## Discussion

The findings of this 4-year follow-up study indicated that exposure to ≥ 3 ACEs (without distinguishing dimensions) increased the risk of developing sarcopenia and that as the number of ACEs increased, the risk also increased. Furthermore, both exposure to ≥ 2 threat- or deprivation-related ACEs independently increased the risk of developing sarcopenia. These findings further indicated that social participation could decrease the risk of developing sarcopenia, with engagement in two or more types of social participation modifying the association between all ACEs (and threat-related ACEs) and developing sarcopenia.

Although studies on the association of ACEs with subsequent illnesses have increased in recent years [4, 5], including chronic diseases [7], dementia [25], and depression [26]), no research has investigated the relationships between ACEs and sarcopenia. However, previous studies have indicated that individuals exposed to ACEs might be at high risk of frailty. A cross-sectional study [27] in Canada involving 27,748 participants aged 45 to 85 years showed that individuals exposed to ACEs had higher frailty index (FI) than those unexposed. Another cross-national study also showed the same result [28]. This cross-national study compared the differences in the relationship between ACEs and the FI between European countries and China. Although the impact of ACEs on the FI varied by countries, there was a common trend that cumulative ACE scores were positively correlated with the FI. Sarcopenia and frailty are both common diseases among older adults and share some common causes, additionally, their definitions overlap in terms of physical function and sarcopenia is also considered one of the main components of frailty [29]. It has been concluded that frailty is often one of the adverse outcomes of sarcopenia, which the study further supports to some extent as well as providing further insights for the prevention of frailty caused by ACEs.

Additionally, our study provides a new perspective on sarcopenia by demonstrating the relationships between ACEs, threat-related ACEs, and deprivation-related ACEs and the development of sarcopenia. However, the mechanisms underlying the association between ACEs and the development of sarcopenia remain poorly understood. Exposure to early-life adversity has been associated with inflammation in adults and impairment of neuroendocrine, metabolic, and cardiovascular systems [26, 30–34] along with DNA methylation and telomere length shorting [35]. In addition, children exposed to ACEs are reported to be more likely to develop behavioural issues (such as excessive smoking and sleep disorders) as they grow up [4, 36]. All these factors are associated with the occurrence of sarcopenia [2, 37]. Therefore, our findings regarding the association between ACEs and the development of sarcopenia later in life are physiologically plausible. A cross-sectional study of 7209 participants showed that ACEs were associated with low muscle strength [6], which may also reveal a potential pathway leading to sarcopenia caused by ACEs.

Although it has been confirmed that ACEs have a negative impact on health [5], this impact presents with cross-national differences [28], which may be related to different characteristics of ACEs in different social-cultural backgrounds [28]. For example, among ACEs among older adults in China, physical abuse (30.8%) and parental death (17.6%) have a higher prevalence, while the prevalence of parental separation (0.5%) is lower [7]. However, among the ACEs in older adults in the United Kingdom, the prevalence of physical abuse (3.3%) and parental death (6.4%) is relatively low, while the prevalence of parental separation (7.0%) is relatively high [38]. Nevertheless, the differences are much less notable in some aspects, such as bad relationship with parents, with the prevalence being 23.1% in China [39] and 24.4% in the United Kingdom [38]. Therefore, future studies that consider different social-cultural backgrounds relation to ACEs and sarcopenia are needed.

One study, including 1483 participants in Kashiwa, Japan, showed that social engagement could potentially decrease new-onset sarcopenia risk by influencing multidimensional factors [40]. Another study, including 5289 participants from the CHARLS, demonstrated that social isolation was associated with an increased risk of sarcopenia [16]. Additionally, a recent review [12] concluded that living alone and social isolation are risk factors for sarcopenia. Our results strengthen the conclusion that active social participation is associated with a low risk of sarcopenia. In the present cohort study, the definition of social participation was based on the number of social activities at baseline, which may not represent the overall level over 4 years. However, the socio-economic status and social relationships of the older adults were relatively stable, and the scope and group of social activities were relatively fixed, so there were unlikely to have been significant changes in the level of social participation among the older adults. A study using the CHARLS data showed that in the three waves of surveys conducted in 2011, 2013, and 2015, nearly half of the respondents did not engage in any social activities in all three waves, and the frequency of various types of social participation varied only slightly between the survey waves, with variations ranging from 0.2 to 6.5% [21].

Previous studies [8, 10, 11] have found that support at the community or social level can alleviate health hazards caused by ACEs. This study further confirmed the modifying role of social participation in the association between ACEs, especially threat-related ACEs, and the development of sarcopenia. This can be explained as follows. First, compared to those with limited social participation, engaging in active social participation is associated with lower levels of inflammation [41, 42]. Second, social participation motivates healthy lifestyles through good social relationships [42, 43]). Third, social participation increases opportunities to access tangible resources and health knowledge that help promote health-promoting behaviours and better health, which reduce the risk of psychological stress and mitigate the likelihood of stress-related neuronal changes through increasing interpersonal interaction [21, 43]. However, when distinguishing the dimensions of ACEs, social participation only had a modifying effect on the association between threat-related ACEs and developing sarcopenia. This may be explained by the different sensitivities of people who have experienced threat- and deprivation-related ACEs. Experiencing threat-related ACEs can lead to functional changes in the amygdala and an enhanced emotional response to environmental stimuli, while experiencing deprivation-related ACEs may affect brain networks related to language development and executive function and lead to a rapid decline in cognitive function over time [11, 44]. Therefore, the modifying effects of social participation on ACEs concerning sarcopenia may be more pronounced among those who have experienced threat-related ACEs.

### Strengths and limitations

Our study had several strengths. First, this is the first study to explore the relationship between ACEs and the different dimensions of ACEs and sarcopenia, providing new insights for identifying vulnerable individuals with sarcopenia. Second, the finding of the modifying effects of social participation on those associations further highlights that support from society/the community can alleviate the effects of ACEs. Third, this was a 4-year follow-up study including a large and nationally representative sample size, which enhances generalisability to the middle-aged and older Chinese general population.

Nevertheless, our study had several limitations. First, some participants were excluded due to missing data, which could have introduced selection bias and reduced generalisability. Second, due to the retrospective nature of life history surveys, information bias regarding ACEs cannot be excluded. However, a previous study [45] showed that retrospective measurements of ACEs had good test-retest reliability and provided distinctive and complementary information. Third, due to the lack of further information on ACEs in the CHARLS database, we could not consider the frequency, intensity, and chronicity of ACEs, all of which are related to health outcomes [5]. Therefore, further in-depth and detailed design is needed for future research on the relationship between ACEs and sarcopenia. Fourth, ASM was assessed using an equation rather than DEXA; therefore, the estimates may have been biased. However, the ASM equation has been validated, and agreement between it and DEXA has been found to be strong (R^2^ = 0.90) [17, 18]. Fifth, although we controlled for demographic characteristics and childhood socioeconomic status-related factors in the models, other unmeasured confounding factors may have distorted the observed associations. Additionally, four years of follow-up might be too short for observing the incident of sarcopenia, and longer follow-up cohort study are needed to confirm this relationship in the future.

## Conclusions

This cohort study’s findings indicated that exposure to ACEs without distinguishing dimensions was associated with the development of sarcopenia among older Chinese adults and that both threat- and deprivation-related factors were independently associated with the development of sarcopenia. Additionally, active social participation may be a significant modifier of the relationship between sarcopenia and ACEs, especially threat-related ACEs. Our findings provide new insights for identifying vulnerable individuals with sarcopenia and emphasise the potential benefits of social participation in the prevention and intervention of sarcopenia in the presence of ACEs. However, further randomised clinical trials and medical experiments are needed to confirm these conclusions and identify the underlying mechanisms behind these relationships.

### Electronic supplementary material

Below is the link to the electronic supplementary material.

Supplementary Material 1

## Data Availability

The datasets generated during and/or analysed during the current study are available in the CHARLS repository, http://charls.pku.edu.cn/.

## Abbreviations

ACEs: adverse childhood experiences
HR: hazard ratio
OR: odds ratio
CI: confidence interval
ASM: appendicular skeletal muscle mass
CHALRS: China Health and Retirement Longitudinal Study
DEXA: dual-energy X-ray absorptiometry
FI: frailty index
