# Supplementary material for: Adverse childhood experiences, sarcopenia, and social participation in older adults: a cohort study
Source: BMC Public Health. 2024 Mar 5;24:711. doi: 10.1186/s12889-024-18138-0 (PMC10916056; doi:10.1186/s12889-024-18138-0)
Supplement: Supplementary file 1 — Supplementary Material 1 [file 12889_2024_18138_MOESM1_ESM.docx]

**Adverse Childhood Experiences, Sarcopenia, and Social Participation in Older Adults: A Cohort Study**

Runnian Huang^1,2^, Yi Li ^2^, Chunhua Ma^2^, Rui Ren^2^, Xiaoyue Yuan^2^, Yang Peng^1*^, and Difei Wang^1*^

^1^ Department of Gerontology and Geriatrics, Shengjing Hospital of China Medical University, Shenyang, Liaoning 110004, China.

^2^ Department of Health Statistics, School of Public Health, China Medical University, Shenyang, Liaoning 110122, China.

* Correspondence: dfwang@cmu.edu.cn; [pengy@sj-hospital.org](mailto:pengy@sj-hospital.org).

***eMethods.*** Participant selection, Appendicular skeletal muscle mass equation and cutoff points, and definition of adverse childhood experience (ACE) items.

*Participant selection*

The baseline data of this research used the China Health and Retirement Longitudinal Study (CHARLS) 2011 baseline survey data. Information regarding ACEs was obtained from the CHARLS 2014 Life History Survey.

A total of 17705 and 20452 individuals were recruited for the CHARLS 2011 baseline survey and the 2014 Life History Survey, respectively. Using ID information, we first conducted a 1:1 matching of the 14434 participants who completed both surveys. We excluded 314 participants aged <45 years or without age information, 2699 participants with missing data on the diagnosis of sarcopenia at baseline, 2467 participants with missing data on any ACE item, and 125 participants with missing data on other variables. Considering that appendicular skeletal muscle mass (ASM) is calculated through an equation including age, sex, height, and weight, to avoid the impact of extreme outliers on calculated ASM, we also excluded 150 participants with height(cm) or weight(kg) exceeding the mean range ± 3*standard deviations, respectively.

A total of 8679 participants were included in baseline analyses. After the further exclusion of 1268 patients with sarcopenia at baseline and 552 participants with missing data on the diagnosis of sarcopenia in 2013 and 2015, 6859 participants were finally included in the main analyses (**eFig.1**).

*Appendicular skeletal muscle mass (ASM) equation and cutoff points*

According to previous research (35059617, 36720206, 22094840, and 27896949), ASM was estimated using a validated anthropometric equation for Chinese residents. Using dual-energy X-ray absorptiometry (DEXA) as the gold standard, the adjusted R^2^ of the model was 0.90 [1, 2].

$$ASM=0.193*weight\left( kg \right)+0.107*height\left( cm \right)-4.157*sex-0.037*age-2.631$$

Height, weight, and age were measured in cm, kg, and years, respectively. For sex, a value of 1 represented male participants, and a value of 2 represented female participants. The cutoff for defining low muscle mass was based on the sex-specific lowest 20% of the height-adjusted muscle mass (ASM/Height^2^) in the study population [1-3]. In this study, the cutoff points were 7.05(Male)/5.36(Female) at the 2011 baseline. In the longitudinal analyses, after excluding patients, the cutoff points were 7.24(Male)/5.66(Female) and 7.30(Male)/5.73(Female) in 2013 and 2015, respectively.

*Definition of ACE items*

In relation to the CHARLS 2014 life history survey, we collected information on ACEs among participants prior to age 17 through face-to-face interviews, and based on previous research [4-6], we extracted 10 ACE items and divided them into two dimensions (eTable 1).

eTable 1: Questionnaire items for adverse childhood events (ACEs)

| Dimensions | | ACE items | Questionnaire items | Definitions |
| --- | --- | --- | --- | --- |
| Total | Threat-related ACEs | Physical abuse | When you were growing up, did your female/male guardian ever hit you? | 0: rarely or never  1: often or sometimes |
|  |  | Household substance abuse | During the years you were growing up, did your female/male guardian ever have alcoholism or drug-related issues? | 0: no  1: yes |
|  |  | Domestic violence | Did your father/mother ever beat up your mother/father? | 0: not very often or never  1: often or sometimes |
|  |  | Unsafe neighbourhood | Was it safe being out alone at night in the neighbourhood where you lived as a child? | 0: very safe or somewhat safe  1: not very safe or not safe |
|  |  | Bullying | 1) When you were a child, how often were you picked on or bullied by kids in your neighbourhood?  2) When you were a child, how often were you picked on or bullied by kids in your school? | 0: not very often or never  1: often or sometimes |
|  | Deprivation-related ACEs | Emotional neglect | 1) How much love and affection did your female guardian give you while you were growing up?  2) How much effort did your female guardian put into watching over you? | 0: often or sometimes  1: rarely or never  0: a lot or some  1: a little or not at all |
|  |  | Household mental illness | 1) Did your female/male guardian have abnormality of mind when you were young?  2) During the years you were growing up, had your female/male guardian often showed continued signs of sadness or depression? | 0: no  1: yes  0: some or only a little of the childhood  1: during all or most |
|  |  | Incarcerated household member | During the years you were growing up, was your female/male guardian  ever arrested or sent to prison? | 0: no  1: yes |
|  |  | Parental separation or divorce | Were your biological parents divorced (including a long separation due to  emotional problems) before you were 17 years old? | 0: no  1: yes |
|  |  | Parental death | Either of the parents was dead before the participant was 17 years old? (Calculated based on the birth date and parental death) | 0: no  1: yes |

Notes: 0 represents absent; 1 represents present.

eTable 2: Characteristics of participants at baseline grouped by adverse childhood events (ACEs) at baseline

|  | 0 | 1 | 2 | ≥3 | p | p. trend |
| --- | --- | --- | --- | --- | --- | --- |
|  | N=2507 | N=2947 | N=1885 | N=1340 |  |  |
| Age | 57.0[50.0;63.0] | 57.0[51.0;64.0] | 57.0[51.0;64.0] | 58.0[51.8;65.0] | <0.001 | <0.001 |
| Sex: |  |  |  |  | <0.001 | <0.001 |
| Male | 1092(43.6%) | 1425(48.4%) | 937(49.7%) | 684(51.0%) |  |  |
| Female | 1415(56.4%) | 1522(51.6%) | 948(50.3%) | 656(49.0%) |  |  |
| Ethnicity: |  |  |  |  | 0.025 | 0.022 |
| Han | 2309(92.1%) | 2739(92.9%) | 1740(92.3%) | 1268(94.6%) |  |  |
| Minority | 198(7.9%) | 208(7.1%) | 145(7.7%) | 72(5.4%) |  |  |
| Childhood residence: |  |  |  |  | 0.013 | 0.023 |
| City/town | 2278(90.9%) | 2707(91.9%) | 1764(93.6%) | 1233(92.0%) |  |  |
| Village | 229(9.1%) | 240(8.1%) | 121(6.4%) | 107(8.0%) |  |  |
| Parental education level: |  |  |  |  | 0.056 | 0.060 |
| Illiterate | 1412(56.3%) | 1678(56.9%) | 1113(59.0%) | 811(60.5%) |  |  |
| Primary school | 906(36.1%) | 1048(35.6%) | 619(32.8%) | 422(31.5%) |  |  |
| Middle school and above | 189(7.5%) | 221(7.5%) | 153(8.1%) | 107(8.0%) |  |  |
| Social participation: |  |  |  |  | 0.544 | 0.072 |
| None | 1225(48.9%) | 1452(49.3%) | 969(51.4%) | 683(51.0%) |  |  |
| One | 846(33.7%) | 986(33.5%) | 599(31.8%) | 446(33.3%) |  |  |
| Two and more | 436(17.4%) | 509(17.3%) | 317(16.8%) | 211(15.7%) |  |  |
| Sarcopenia: |  |  |  |  | <0.001 | <0.001 |
| No | 2195(87.6%) | 2528(85.8%) | 1592(84.5%) | 1096(81.8%) |  |  |
| Yes | 312(12.4%) | 419(14.2%) | 293(15.5%) | 244(18.2%) |  |  |

Notes: The percentages of polytomous variables may not sum up to 100% because of rounding.

eTable 3: Baseline characteristics of participants grouped by threat-related adverse childhood events (ACEs) at baseline

|  | 0 | 1 | ≥2 | p | p. trend |
| --- | --- | --- | --- | --- | --- |
|  | N=4647 | N=2610 | N=1422 |  |  |
| Age | 58.0[51.0;64.0] | 57.0[50.0;64.0] | 56.0[50.0;63.0] | <0.001 | <0.001 |
| Sex: |  |  |  | <0.001 | <0.001 |
| Male | 2020(43.5%) | 1331(51.0%) | 787(55.3%) |  |  |
| Female | 2627(56.5%) | 1279(49.0%) | 635(44.7%) |  |  |
| Ethnicity: |  |  |  | 0.326 | 0.174 |
| Han | 4302(92.6%) | 2421(92.8%) | 1333(93.7%) |  |  |
| Minority | 345(7.4%) | 189(7.2%) | 89(6.3%) |  |  |
| Childhood residence: |  |  |  | 0.026 | 0.155 |
| City/town | 4245(91.3%) | 2431(93.1%) | 1306(91.8%) |  |  |
| Village | 402(8.7%) | 179(6.9%) | 116(8.2%) |  |  |
| Parental education level: |  |  |  | 0.227 | 0.446 |
| Illiterate | 2683(57.7%) | 1505(57.7%) | 826(58.1%) |  |  |
| Primary school | 1631(35.1%) | 892(34.2%) | 472(33.2%) |  |  |
| Middle school and above | 333(7.2%) | 213(8.2%) | 124(8.7%) |  |  |
| Social participation: |  |  |  | 0.153 | 0.705 |
| None | 2314(49.8%) | 1287(49.3%) | 728(51.2%) |  |  |
| One | 1579(34.0%) | 853(32.7%) | 445(31.3%) |  |  |
| Two and more | 754(16.2%) | 470(18.0%) | 249(17.5%) |  |  |
| Sarcopenia: |  |  |  | 0.958 | 0.941 |
| No | 3969(85.4%) | 2225(85.2%) | 1217(85.6%) |  |  |
| Yes | 678(14.6%) | 385(14.8%) | 205(14.4%) |  |  |
| Deprivation-related ACEs: |  |  |  | <0.001 | <0.001 |
| None | 2507(53.9%) | 1226(47.0%) | 558(39.2%) |  |  |
| One | 1721(37.0%) | 1066(40.8%) | 617(43.4%) |  |  |
| Two and more | 419(9.0%) | 318(12.2%) | 247(17.4%) |  |  |

Notes: The percentages of polytomous variables may not sum up to 100% because of rounding.

eTable 4: Baseline characteristics of participants grouped by deprivation-related adverse childhood events (ACEs) at baseline

|  | 0 | 1 | ≥2 | p | p. trend |
| --- | --- | --- | --- | --- | --- |
|  | N=4291 | N=3404 | N=984 |  |  |
| Age | 56.0[50.0;62.5] | 58.0[51.0;64.0] | 60.0[54.0;67.0] | <0.001 | <0.001 |
| Sex: |  |  |  | 0.018 | 0.005 |
| Male | 2108(49.1%) | 1588(46.7%) | 442(44.9%) |  |  |
| Female | 2183(50.9%) | 1816(53.3%) | 542(55.1%) |  |  |
| Ethnicity: |  |  |  | 0.011 | 0.034 |
| Han | 3949(92.0%) | 3193(93.8%) | 914(92.9%) |  |  |
| Minority | 342(8.0%) | 211(6.2%) | 70(7.1%) |  |  |
| Childhood residence: |  |  |  | 0.089 | 0.052 |
| City/town | 3930(91.6%) | 3130(92.0%) | 922(93.7%) |  |  |
| Village | 361(8.4%) | 274(8.1%) | 62(6.3%) |  |  |
| Parental education level: |  |  |  | <0.001 | <0.001 |
| Illiterate | 2412(56.2%) | 1963(57.7%) | 639(64.9%) |  |  |
| Primary school | 1538(35.8%) | 1177(34.6%) | 280(28.5%) |  |  |
| Middle school and above | 341(8.0%) | 264(7.8%) | 65(6.6%) |  |  |
| Social participation: |  |  |  | 0.006 | 0.001 |
| None | 2087(48.6%) | 1728(50.8%) | 514(52.2%) |  |  |
| One | 1421(33.1%) | 1121(32.9%) | 335(34.0%) |  |  |
| Two and more | 783(18.2%) | 555(16.3%) | 135(13.7%) |  |  |
| Sarcopenia: |  |  |  | <0.001 | <0.001 |
| No | 3756(87.5%) | 2899(85.2%) | 756(76.8%) |  |  |
| Yes | 535(12.5%) | 505(14.8%) | 228(23.2%) |  |  |
| Threat-related ACEs: |  |  |  | <0.001 | <0.001 |
| None | 2507(58.4%) | 1721(50.6%) | 419(42.6%) |  |  |
| One | 1226(28.6%) | 1066(31.3%) | 318(32.3%) |  |  |
| Two and more | 558(13.0%) | 617(18.1%) | 247(25.1%) |  |  |

Notes: The percentages of polytomous variables may not sum up to 100% because of rounding.

eTable 5: Associations between ACEs and sarcopenia at baseline

| ACEs | OR (95%CI) |  |
| --- | --- | --- |
|  | Crude model | Adjusted model |
| ACE group |  |  |
| 0 | ref | ref |
| 1 | 1.17 (1.00,1.37) | 1.09 (0.92,1.30) |
| 2 | 1.29 (1.09,1.54) | 1.14 (0.94,1.39) |
| ≥3 | 1.57 (1.30,1.88) | 1.39 (1.13,1.70) |
| Number of ACEs | 1.12 (1.07,1.18) | 1.09 (1.03,1.15) |
| Threat-related ACEs |  |  |
| 0 | ref | ref |
| 1 | 1.01 (0.88,1.16) | 1.01 (0.86,1.17) |
| ≥2 | 0.99 (0.83,1.17) | 1.07 (0.88,1.30) |
| Deprivation-related ACEs |  |  |
| 0 | ref | ref |
| 1 | 1.22 (1.07,1.39) | 1.06 (0.92,1.23) |
| ≥2 | 2.12 (1.78,2.52) | 1.49 (1.22,1.81) |

Notes: Models were adjusted for age, sex, ethnicity, childhood residence, and educational level of parents, and when distinguishing dimensions, additionally adjusted for the two dimensions.

Abbreviation: ACE, adverse childhood experience; OR, odds ratio; CI, confidence interval.

eTable 6: Association of social participation and sarcopenia at baseline

| Social participation | OR (95%CI) |  |
| --- | --- | --- |
|  | Crude model | Adjusted model |
| None | ref | ref |
| One | 0.78 (0.68,0.89) | 0.75 (0.64,0.87) |
| Two and more | 0.43 (0.35,0.52) | 0.53 (0.42,0.66) |
| Number of social participation | 0.70 (0.64,0.76) | 0.74 (0.68,0.81) |

Notes: Models were adjusted for age, sex, ethnicity, childhood residence, and the educational level of parents.

Abbreviations: OR, odds ratio; CI, confidence interval

eTable 7: The characteristics of participants grouped according to the occurrence of sarcopenia within four years of follow-up

|  | No | Yes | p |
| --- | --- | --- | --- |
|  | N=5680 | N=1179 |  |
| Age | 55.0 [49.0;60.0] | 61.0 [57.0;66.0] | <0.001 |
| Sex: |  |  | 0.537 |
| Male | 2699 (47.5%) | 548 (46.5%) |  |
| Female | 2981 (52.5%) | 631 (53.5%) |  |
| Ethnicity: |  |  | 0.319 |
| Han | 5309 (93.5%) | 1092 (92.6%) |  |
| Minority | 371 (6.5%) | 87 (7.4%) |  |
| Childhood residence: |  |  | 0.001 |
| City/town | 5183(91.2%) | 1112 (94.3%) |  |
| Village | 497 (8.8%) | 67 (5.7%) |  |
| Parental education level: |  |  | <0.001 |
| Illiterate | 3100 (54.6%) | 738 (62.6%) |  |
| Primary school | 2060 (36.3%) | 382 (32.4%) |  |
| Middle school and above | 520 (9.2%) | 59 (5.0%) |  |
| Social participation: |  |  | <0.001 |
| None | 2668 (47.0%) | 644 (54.6%) |  |
| One | 1922 (33.8%) | 390 (33.1%) |  |
| Two and more | 1090 (19.2%) | 145 (12.3%) |  |
| ACE group: |  |  | 0.004 |
| 0 | 1710 (30.1%) | 322 (27.3%) |  |
| 1 | 1956 (34.4%) | 388 (32.9%) |  |
| 2 | 1216 (21.4%) | 258 (21.9%) |  |
| ≥3 | 798 (14.0%) | 211 (17.9%) |  |

Notes: The percentages of polytomous variables may not sum up to 100% because of rounding.

Abbreviation: ACE, adverse childhood experience

eTable 8: Hazard ratios (HRs) of social participation in developing sarcopenia within four years of follow-up

| Social participation | HR (95%CI) |  |
| --- | --- | --- |
|  | Crude model | Adjusted model |
| None | ref | ref |
| One | 0.87 (0.76,0.98) | 0.85 (0.75,0.97) |
| Two and more | 0.58 (0.49,0.70) | 0.68 (0.57,0.82) |
| Number of social participation | 0.80 (0.75,0.86) | 0.84 (0.78,0.91) |

Notes: Models were adjusted for age, sex, ethnicity, childhood residence, and the educational level of parents.

Abbreviations: CI, confidence interval

eTable 9: The relationships between ACEs and social participation.

| ACEs | Beta | p |
| --- | --- | --- |
| ACE group |  |  |
| 0 | ref |  |
| 1 | 0.013 | 0.637 |
| 2 | 0.013 | 0.659 |
| ≥3 | -0.024 | 0.483 |
| Number of ACEs | -0.006 | 0.476 |
| Threat-related ACEs |  |  |
| 0 | ref |  |
| 1 | 0.037 | 0.139 |
| ≥2 | 0.019 | 0.543 |
| Deprivation-related ACEs |  |  |
| 0 | ref |  |
| 1 | -0.034 | 0.135 |
| ≥2 | -0.050 | 0.178 |

Notes: Models were adjusted for age, sex, ethnicity, childhood residence, and educational level of parents, and when distinguishing dimensions, additionally adjusted for the two dimensions.

Abbreviation: ACE, adverse childhood experience; OR, odds ratio; CI, confidence interval

**eFig.1** Flowchart of the participants


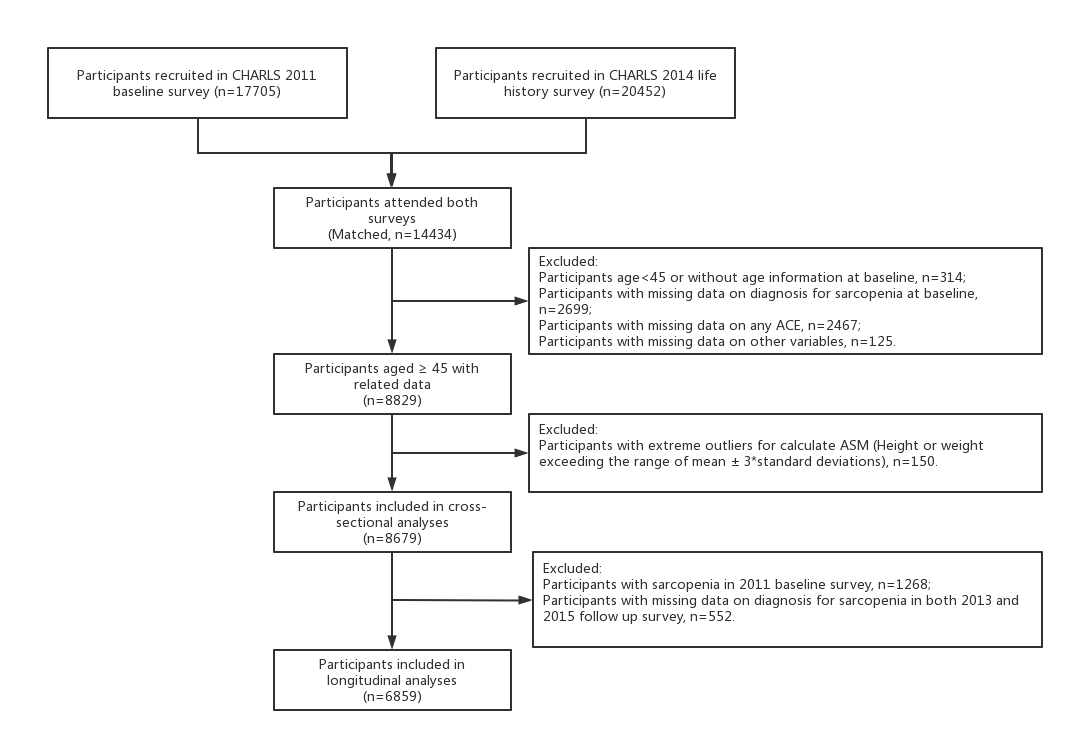


**eFig.2** Prevalence of sarcopenia at baseline


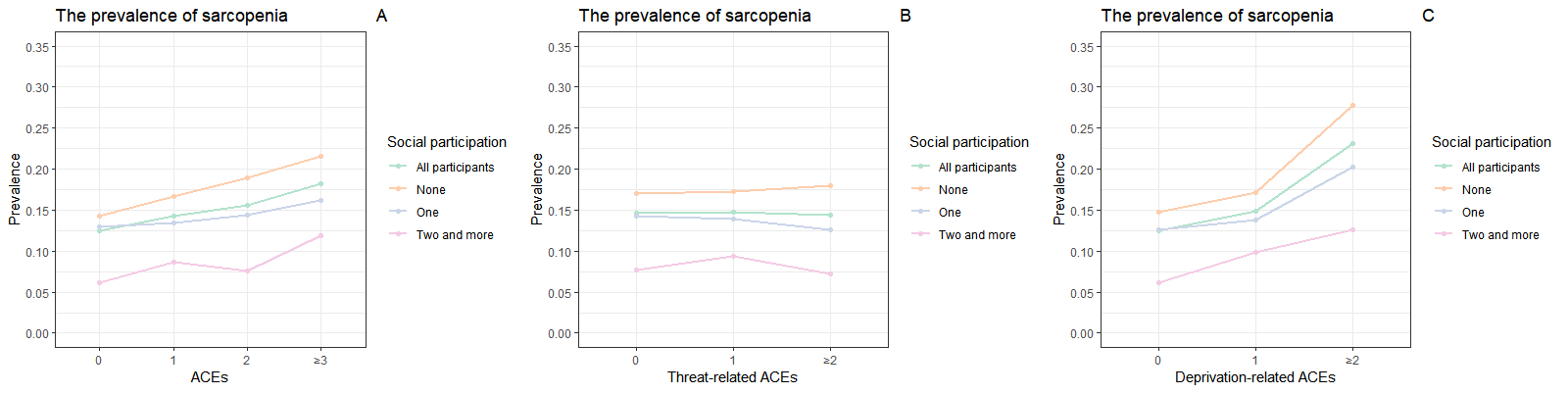


Abbreviations: ACE, adverse childhood experience

**eFig.3** Incidence of sarcopenia within four years of follow-up


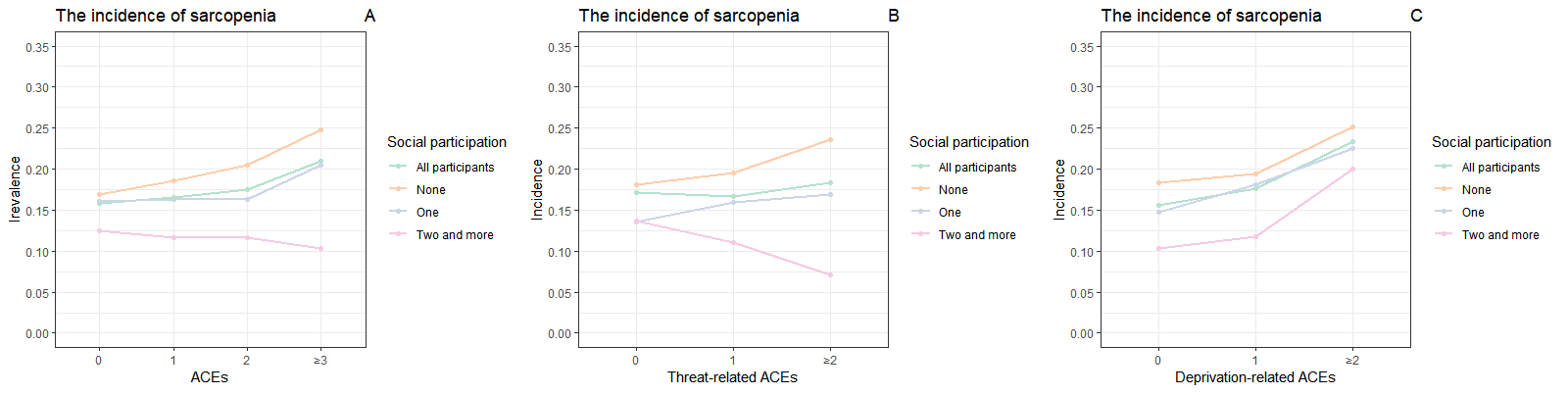


Abbreviations: ACE, adverse childhood experience

**eFig.4** Relationship between deprivation-related adverse childhood events (ACEs) and development of sarcopenia by subgroup analysis in terms of social participation within four years of follow-up


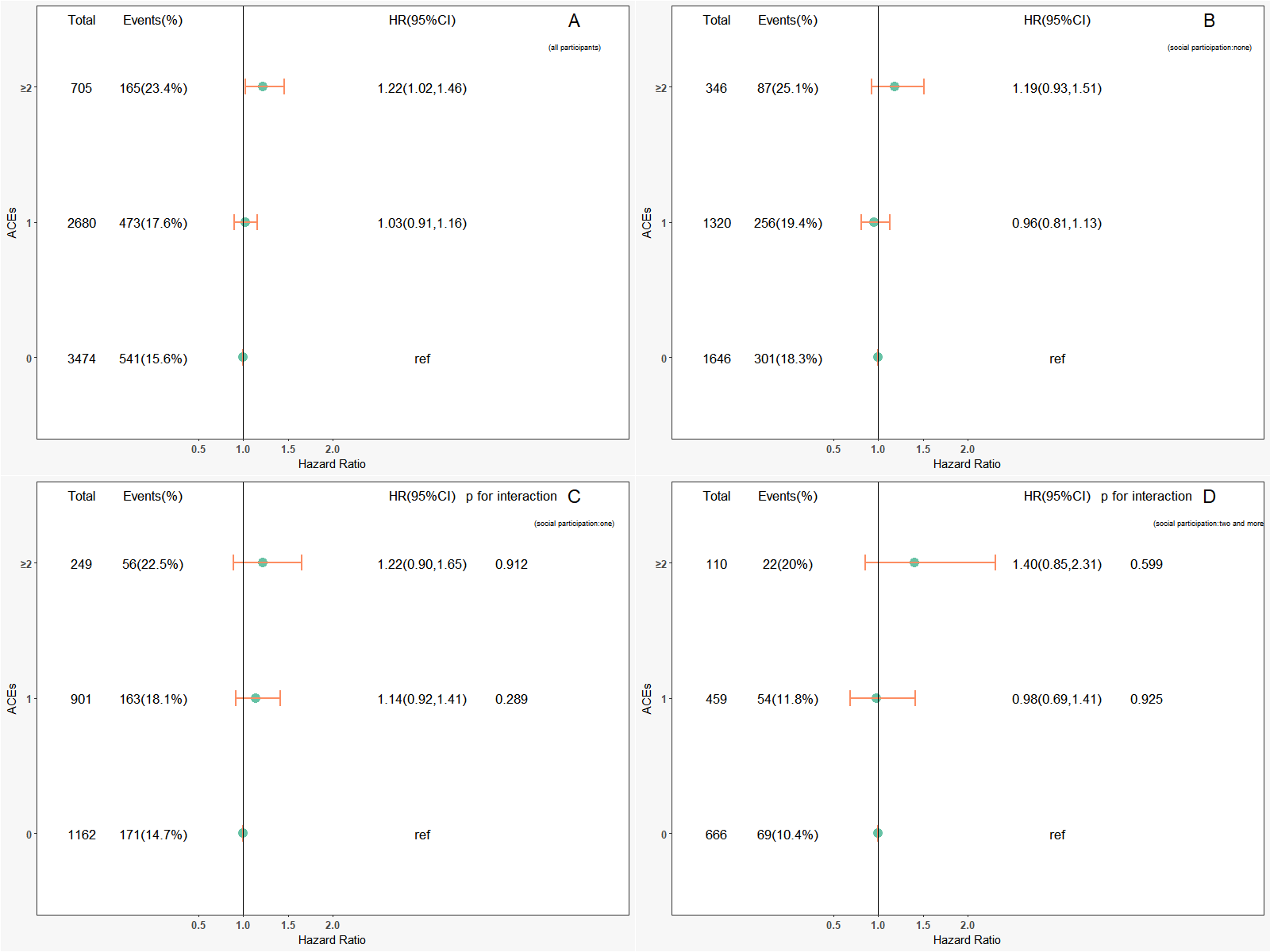


A: all participants, B: social participation=none, C: social participation=one; D: social participation= two and more.

Abbreviations: HR, hazard ratio, CI, confidence interval

**References**

[1] Wen X, Wang M, Jiang CM, Zhang YM. Anthropometric equation for estimation of appendicular skeletal muscle mass in Chinese adults. Asia Pacific journal of clinical nutrition. 2011;20:551-6.

[2] Yang M, Hu X, Wang H, Zhang L, Hao Q, Dong B. Sarcopenia predicts readmission and mortality in elderly patients in acute care wards: a prospective study. Journal of cachexia, sarcopenia and muscle. 2017;8:251-8.

[3] Gao K, Cao LF, Ma WZ, Gao YJ, Luo MS, Zhu J, et al. Association between sarcopenia and cardiovascular disease among middle-aged and older adults: Findings from the China health and retirement longitudinal study. EClinicalMedicine. 2022;44:101264.

[4] Lin L, Wang HH, Lu C, Chen W, Guo VY. Adverse Childhood Experiences and Subsequent Chronic Diseases Among Middle-aged or Older Adults in China and Associations With Demographic and Socioeconomic Characteristics. JAMA network open. 2021;4:e2130143.

[5] Wolf S, Suntheimer NM. A dimensional risk approach to assessing early adversity in a national sample. Journal of Applied Developmental Psychology. 2019;62:270-81.

[6] Lin L, Cao B, Chen W, Li J, Zhang Y, Guo VY. Association of Adverse Childhood Experiences and Social Isolation With Later-Life Cognitive Function Among Adults in China. JAMA network open. 2022;5:e2241714.
